# Serological evidence for potential yellow fever virus infection in non-human primates, southeastern Mexico

**DOI:** 10.1186/s42522-023-00090-5

**Published:** 2023-10-24

**Authors:** Mónica Salas-Rojas, Edmilson Ferreira de Oliveira-Filho, Cenia Almazán-Marín, Alba Zulema Rodas-Martínez, Álvaro Aguilar-Setién, Jan Felix Drexler

**Affiliations:** 1 UIM en Inmunología, UMAE Hospital de Pediatría, Centro Médico Nacional “Siglo XXI”, Instituto Mexicano del Seguro Social, Ciudad de Mexico, México; 2 Institute of Virology, Charité-Universitätsmedizin Berlin, corporate member of Freie Universität Berlin and Humboldt-Universität Zu Berlin, Charitéplatz 1, 10117 Berlin, Germany; 3 División Académica de Ciencias Biológicas, Universidad Juárez Autónoma de Tabasco, Villahermosa, Tabasco México; 4 German Centre for Infection Research (DZIF), Associated Partner Site Charité, Berlin, Germany

**Keywords:** Yellow fever virus, Flavivirus, *Alouatta*, *Ateles*, Antibodies

## Abstract

**Background:**

Arthropod-borne flaviviruses like dengue virus (DENV) and yellow fever virus (YFV) are major human pathogens. In Latin America, YFV is maintained in sylvatic cycles involving non-human primates (NHP) and forest-dwelling mosquitos. YFV supposedly does not circulate north of Panama.

**Methods:**

We conducted a serologic study for flaviviruses and other emerging viruses in NHP from southeastern Mexico. A total of thirty sera of black-handed spider monkeys (*Ateles geoffroyi*, *n* = 25), black howler monkeys (*Alouatta pigra*, *n* = 3), and mantled howler monkeys (*Al. palliata*, *n* = 2) sampled in 2012 and 2018 were screened by an indirect immunofluorescence assay (IFA) to detected IgG antibodies against DENV, YFV, Zika virus (ZIKV), West Nile virus (WNV), Rift Valley fever virus, Crimean-Congo hemorrhagic fever virus, Middle East respiratory syndrome coronavirus, and Zaire Ebola virus, and confirmed by plaque reduction neutralization tests (PRNT_90_) representing all mosquito-borne flavivirus serocomplexes circulating in the Americas.

**Results:**

A total of 16 sera (53.3%; 95% CI, 34.3–71.7) showed IFA reactivity to at least one tested flavivirus with end-point titers ranging from 1:100 to 1:1000. No serum reacted with other viruses. Monotypic and high mean PRNT_90_ endpoint YFV titers of 1:246 were found in 3 black-handed spider monkey sera (10.0%; 95% CI, 2.1–26.5) sampled in 2018 in Tabasco, compared to all other flaviviruses tested. Monotypic endpoint PRNT_90_ titers of 1:28 for Ilheus virus and 1:22 for WNV in serum of black howler monkeys sampled in 2018 in Tabasco suggested additional flavivirus exposure.

**Conclusions:**

Our findings may suggest unnoticed YFV circulation. Intensification of YFV surveillance in NHP and vectors is warranted in Mexico and potentially other areas considered free of yellow fever.

## Background

**Fig. 1:**
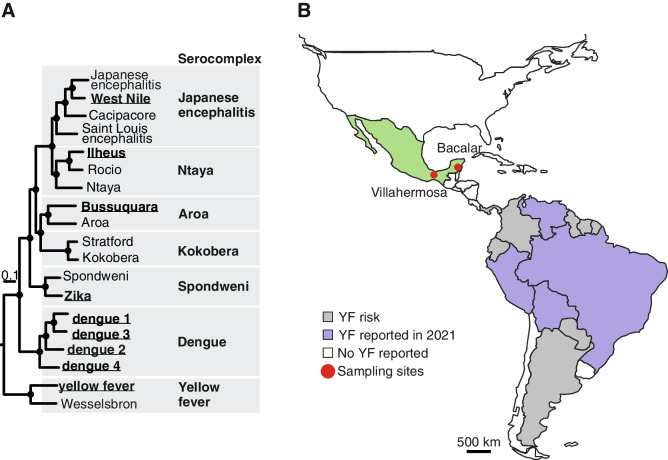
Flavivirus serocomplexes and sampling sites. **A** Countries in Central and South America that reported yellow fever (YF) cases in 2021 or are at risk of yellow fever outbreaks, and collection area in Mexico [7, 8]. **B** Phylogeny of selected mosquito-borne flaviviruses was calculated in MrBayes (v3.2.6; 20 000 trees from 2 000 000 generations using the translated polyprotein gene and a Whelan and Goldman substitution model; black dots indicate posterior probability > 0∙9; Nienokoue virus was used as an outgroup). Viruses that were included in the study are indicated in bold and underlined

Flaviviruses are widespread in almost all continents and climate zones [1]. In Latin America, flavivirus infections have caused an enormous impact on public health, with dengue virus (DENV) infecting millions of people yearly, yellow fever virus (YFV) causing several outbreaks, and most recently with the emergence of West Nile virus (WNV) and Zika virus (ZIKV) [1–4]. Mosquito-borne flaviviruses can be grouped into seven serocomplexes (Aroa, Dengue, Kokobera, Spondweni, Japanese encephalitis, Yellow Fever, Ntaya) [5, 6] (Fig. 1A), with three (Dengue, Spondweni, and Japanese encephalitis) of them reported in Mexico.

In Mexico, dengue fever is endemic, with 25,226 cases reported in 2020 and 6746 in 2021 [9]. WNV was first reported in Mexico in 2002 in birds, horses, humans and *Culex* mosquitos at different times and places [7]. ZIKV was introduced in the country in 2015, and 11,869 cases have been reported since [9]. In contrast to those three endemic flaviviruses, YFV was present in the country from the XIX century until 1963 only, when the country was considered free of yellow fever thanks to extensive campaigns to eliminate the urban vector *Aedes aegypti* [8]. In 2021, according to the Pan American Health Organization (PAHO), four countries reported yellow fever cases in South America (659 reported / 31 confirmed), and 13 countries in Central and South America were considered at risk, ending northwards with Panama (Fig. 1B) [10, 11]. Typically, only severe cases of yellow fever are identified, and given that approximately 90% of cases are mild or exhibit no symptoms, this may contribute to the lack of reported cases [12].

With circa 200,000 cases and 30,000 deaths annually, YFV is currently endemic in 47 countries in Africa, Central and South America [13, 14]. Urban and sylvatic flavivirus transmission cycles can occur independently or in parallel. Sylvatic cycles of YFV involving non-human primates (NHP) nowadays prevail in the New World [15, 16]. YFV infection in New World NHP is very severe and can be fatal, however disease severity is variable among different NHP species [17]. Sylvatic YFV cycles involve mosquitos of the genera *Haemagogus* and *Sabethes* which have been reported in Southeastern Mexico [18, 19]. Because the urban YFV vectors, *Aedes* mosquitoes, are widely distributed throughout the country, the re-emergence of YFV in urban regions could have devastating consequences. Moreover, because YFV infection is potentially deadly for primates and the three NHP species occurring in Mexico are listed as vulnerable (*Alouatta palliata*) and endangered (*Al. pigra* and *Ateles geoffroyi*), the potential presence of YFV in Mexican NHP may pose a great risk for animal conservation. Here we investigated the presence of flaviviruses in NHP from the southeast of Mexico.

## Methods

We tested a total of thirty NHP sera, twenty-one samples were from Bacalar, Quintana Roo and nine from Villahermosa, Tabasco (both sites in southeastern Mexico); twenty-five *Ateles geoffroyi* (black-handed spider monkey), three *Alouatta pigra* (black howler monkey), and two *Al. palliata* (mantled howler monkey). The samples from Quintana Roo (2012) were obtained from animals that were part of a wildlife translocation program at a government airport, in which samples were taken from the animals before being relocated and sent to our laboratories for the detection of viral infectious diseases, before their release. The animals were kept in a temporary shelter located in Km 20 + 500 Carretera Federal 307, tramo Bacalar-Carrillo Puerto, Quintana Roo. The samples from Tabasco (2018) belonged to captive monkeys at state zoological facilities La Venta Park-Museum and Yumká Natural Protected Area (18°00′02″N 92°56′08″O, and 17°59′22″N 92°55′41″O; respectively). Because of the importance of monitoring potentially zoonotic diseases, authorities of both institutions authorized the physical restraint of individuals, which is a technique considered appropriate in captive primates for procedures of short duration. Animal handling was performed by specialized and experienced staff, following protocols of safety, care, and health of each institution. All animals were in good overall conditions at the time of blood sampling (2 – 3 mL from the coccygeal vein for each specimen) and samples were stored on ice and immediately transported to the laboratory. Serum samples from all NHP that were available at the two locations were tested, without application of additional inclusion or exclusion criteria.

To detect IgG antibodies against selected members of the *Phenuiviridae*, *Flaviviridae*, *Coronaviridae*, and *Filoviridae* families, we employed an indirect immunofluorescence test (IFA) termed Biochip Virus-Mosaic Africa-2 (Euroimmun, Germany; FK 280a-1010–2) following the manufacturer’s instructions. The biochip contains antigens from high-risk pathogens such as DENV, WNV, YFV, ZIKV, Rift Valley fever virus, Crimean-Congo hemorrhagic fever virus, Middle East respiratory syndrome coronavirus, and Zaire Ebola virus. Because of the well-described antibody cross-reactivity among flaviviruses, samples that tested positive for at least one flavivirus were further tested for specific viruses by plaque reduction neutralization test (PRNT). PRNT was performed using ZIKV (strain H/PF/2013), DENV-1 (strain Thailand/16007), DENV-2 (strain 16681), DENV-3 (strain Philippines/H87), DENV-4 (strain Philippines/H241), WNV (strain NY-99), YFV (strain 17D), Bussuquara virus (BSQV; strain BeAn 4073), Ilheus virus (ILHV; strain UVE/ILHV/UNK/PE/PE20545). Briefly, sera were inactivated at 56° for 30 min and diluted at 1:80, 1:240, 1:720 and 1:2160 in serum-free DMEM. Subsequently, a mixture of 34 µL of each serum dilution plus 34 µL of virus dilution containing 50 plaque forming units was incubated for 1 h at 37° C with 5% CO_2_. After this time, 50 µL of the serum dilution and virus mixture plus 250 µL of cell culture media were added to 12-well plates containing Vero FM (for ZIKV, WNV, BSQV, ILHV and YFV) and Vero B4 (for DENV1-4) cells seeded one day before (1.4 × 10^5^ cells/well). After one hour of incubation, an overlay containing 2% DMEM and 1.25% carboxymethyl cellulose was added. After three days for WNV, four days for BSQV, five days for ZIKV, six days for ILHV and DENV, and seven days for YFV, the medium was removed, and cells were fixed with 6% paraformaldehyde and plaques visualized using crystal violet solution. A positive result means a serum dilution reduced the number of viral plaques in a given well by at least 50% (PRNT_50_) or 90% (PRNT_90_). Additionally, NHP sera were tested for flaviviruses by semi-nested RT-PCR using primers previously reported [20], using SuperScript™ III One-Step RT-PCR system with Platinum™ *Taq* DNA polymerase (Invitrogen, USA), and *Taq* DNA polymerase (Invitrogen, USA), for the first and second round, respectively, following the manufacturer’s instructions.

## Results

**Fig. 2:**
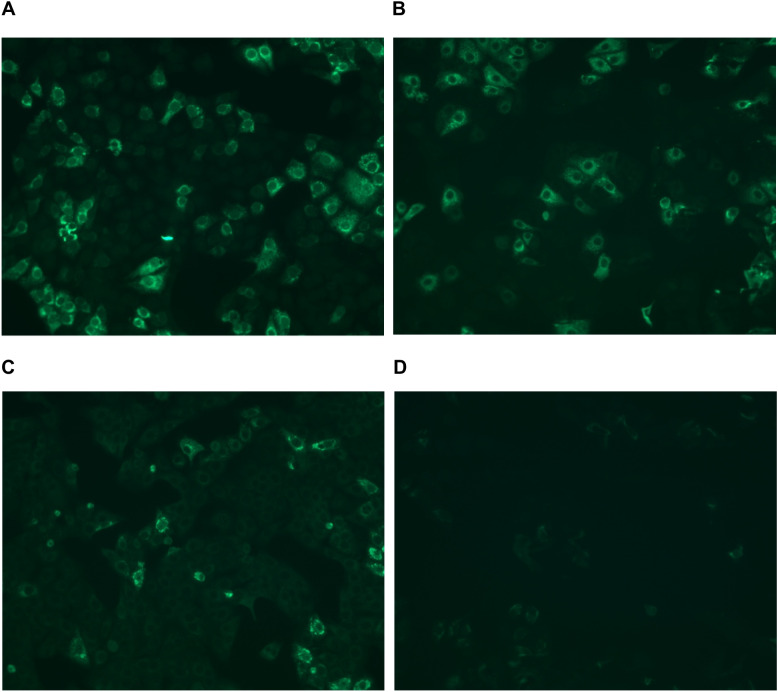
Immunofluorescence assay (IFA) to detect antibodies against flaviviruses. **A** Sample positive to DENV-2 (ID 604); **B** sample positive to WNV (ID 875); **C** sample positive to YFV (ID 879); **D** sample negative to ZIKV (ID 875)

Initial IFA screening showed that 53% (16/30) of sera were reactive for at least one of the flavivirus antigens tested (WNV, ZIKV, DENV, and YFV), with end-point titers ranging from 1:100 to 1:1000 serum dilution, not allowing unambiguous identification of flavivirus infection histories (Table 1; Fig. 2). No sample was positive against other viruses included in the IFA Biochip, suggesting absence of antigenically related phlebo-, filo or coronaviruses in those Mexican NHP and all samples tested negative for flaviviral RNA in the RT-PCR.

**Table 1:** Flavivirus antibody detection using an indirect immunofluorescence assay in sera from non-human primates, Southeastern Mexico

**ID**	**Sampling year**	**State of origin**	**Species**	**IFA (final dilution yielding IgG antibody reactivity)**			
				**WNV**	**ZIKV**	**DENV**	**YFV**
603	2012	Quintana Roo	*Ateles geoffroyi*	1:100	1:100	1:100	1:100
604	2012	Quintana Roo	*At. geoffroyi*	1:1000	1:1000	1:1000	1:1000
605	2012	Quintana Roo	*At. geoffroyi*	1:1000	-	-	-
610	2012	Quintana Roo	*At. geoffroyi*	1:1000	1:1000	1:1000	1:1000
621	2012	Quintana Roo	*At. geoffroyi*	-	1:1000	-	-
623	2012	Quintana Roo	*At. geoffroyi*	-	1:100	-	-
624	2012	Quintana Roo	*At. geoffroyi*	-	1:1000	-	-
873	2018	Tabasco	*Alouatta pigra*	1:100	1:100	-	-
874	2018	Tabasco	*Al. pigra*	-	1:100	-	-
875	2018	Tabasco	*Al. pigra*	1:1000	1:1000	1:1000	1:1000
877	2018	Tabasco	*Al. palliata*	-	1:100	-	-
878	2018	Tabasco	*Al. palliata*	-	1:100	-	-
879	2018	Tabasco	*At. geoffroyi*	1:1000	1:1000	1:1000	1:1000
880	2018	Tabasco	*At. geoffroyi*	1:100	1:100	1:100	1:100
881	2018	Tabasco	*At. geoffroyi*	1:100	-	1:100	1:100
882	2018	Tabasco	*At. geoffroyi*	1:1000	1:1000	1:1000	1:1000

*“-”* not reactive, *IFA* indirect immunofluorescence assay, *YFV* yellow fever virus, *WNV* West Nile virus, *ZIKV* Zika virus, *DENV* dengue virus

**Fig. 3:**
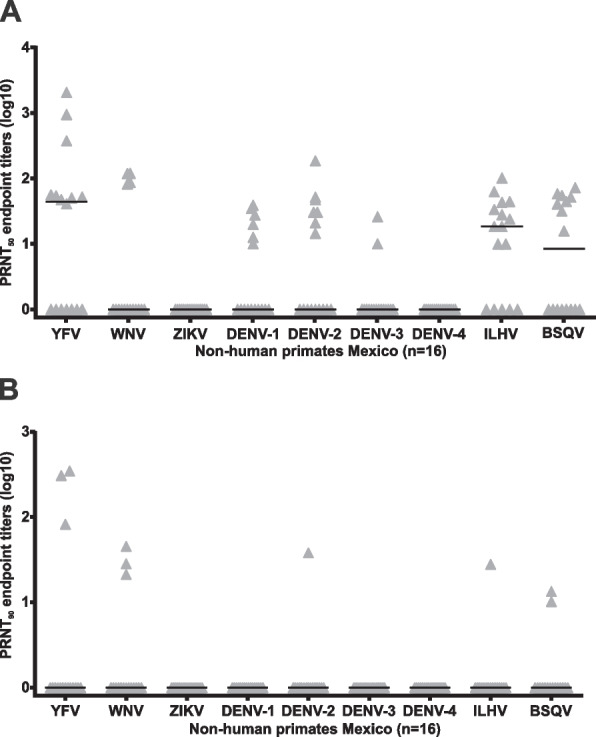
Reciprocal plaque reduction neutralization (PRNT) endpoint titers against selected flaviviruses in sera from Mexican non-human primates. **A** PRNT_50_ and **B** PRNT_90_

Because antibody cross-reactivity is very common among flaviviruses, we tested all IFA-positive sera by highly specific PRNT against at least one flavivirus of each serocomplex. Both PRNT_50_ and PRNT_90_ titers were calculated, but we selected PRNT_90_ to achieve higher specificity levels by reducing the impact of antibody cross-reactivity leading to inconclusive PRNT_50_ results in most sera (Fig. 3A; Table 2). High and monotypic PRNT_90_ titers were found against YFV (mean, 245.9; SD, 142.9; range 82.6–348.0) in 12.5% (3/24; 95% CI, 2.1–26.5) of the captive black-handed spider monkeys sampled in Tabasco in 2018 (Table 2; Fig. 3B). The presence of neutralizing antibodies against YFV in NHP in Mexico is unexpected since, to date, YFV has only been reported outside South America in the 1970ies in Panama and Trinidad and Tobago (Fig. 1B), despite a large outbreak in humans and NHP affecting many countries in South America in 2016–2018 [15, 16, 21]. On the other hand, YFV seropositive NHP lived all their lives or for many years in the same location: animal 879 arrived in the park in 1997 and was sampled after 21 years; animal 881 was born in the park in 2007 and sampled after 11 years and 882 arrived in the park in 2009 and was sampled after 9 years. Moreover the ability of *Ateles* species to elicit an immune response against YFV is supported by previous finding in the 1950s in free-living animals showing the presence of antibodies in black-handed spider monkeys in Panama [22] and in spider monkeys in Brazil [23]. The absence of antibodies in black howler monkeys (genus *Alouatta*) must be interpreted with caution because of the low number of animals tested. However, absence of antibodies in those animals is consistent with high mortality of yellow fever in NHP belonging to *Alouatta* species exceeding 60%, in contrast to NHP species of the genus *Ateles* such as spider monkeys which seem to be more resistant to infection and clinical disease.

**Table 2:** Neutralizing antibody endpoint titers against different flaviviruses in sera from non-human primates, Southeastern Mexico

**Sero-complex**	**JE**		**SPO**		**Dengue**								**YF**		**Ntaya**		**Aroa**		**Interpretation**	
**Viruses**	**WNV**		**ZIKV**		**DENV-1**		**DENV-2**		**DENV-3**		**DENV-4**		**YFV**		**ILHV**		**BSQV**		**PRNT**_**50**_	**PRNT**_**90**_
**PRNT**	50	90	50	90	50	90	50	90	50	90	50	90	50	90	50	90	50	90		
**603**	-	-	-	-	-	-	-	-	-	-	-	-	52.5	-	-	-	-	-	YFV	Inc
**604**	-	-	-	-	10.1	-	187.l	38.2	10.1	-	-	-	54.3	-	-	-	-	-	Inc	DENV-2
**605**	-	-	-	-	-	-	-	-	-	-	-	-	-	-	18.5	-	31.8	-	Inc	Inc
**610**	-	-	-	-	27.9	-	-	-	-	-	-	-	-	-	10.0	-	-	-	Inc	Inc
**621**	-	-	-	-	-	-	-	-	-	-	-	-	-	-	18.5	-	40.9	-	Inc	Inc
**623**	-	-	-	-	-	-	-	-	-	-	-	-	50.4	-	-	-	58.4	10.0	Inc	Inc ^a^
**624**	-	-	-	-	-	-	21.1	-	-	-	-	-	-	-	-	-	15.8	-	Inc	Inc
**873**	-	-	-	-	-	-	30.6	-	-	-	-	-	41.0	-	10.0	-	44.8	-	Inc	Inc
**874**	-	-	-	-	-	-	47.4	-	-	-	-	-	47.4	-	102.0	28.0	-	-	Inc	ILHV ^b^
**875**	108.0	21.6	-	-	12.7	-	-	-	-	-	-	-	-	-	28.1	-	-	-	WNV	WNV
**877**	-	-	-	-	-	-	-	-	-	-	-	-	-	-	44.5	-	72.5	13.0	Inc	Inc ^a^
**878**	-	-	-	-	-	-	-	-	-	-	-	-	-	-	-	-	52.0	-	BSQV	Inc
**879**	83.1	-	-	-	39.0	-	30.6	-	26.1	-	-	-	377.0	82.6	63.0	-	-	-	YFV	YFV
**880**	-	-	-	-	-	-	-	-	-	-	-	-	56.6	-	43.4	-	-	-	Inc	Inc
**881**	120.0	18.5	-	-	35.0	-	14.6	-	-	-	-	-	2066.0	348.0	33.7	-	55.2	-	YFV	YFV
**882**	134.0	24.8	-	-	19.9	-	52.0	-	-	-	-	-	947.0	308.0	24.0	-	-	-	YFV	YFV

*PRNT* plaque reduction neutralization test, *“Inc”* Inconclusive, *“-”* no neutralization observed, *JE* Japanese encephalitis, *SPO* Spondweni, *YF* yellow fever, *YFV* yellow fever virus, *WNV* West Nile virus, *ZIKV* Zika virus, *DENV* dengue virus, *ILHV* Ilheus virus, *BSQV* Bussuquara virus

^a^Although low titers were observed for BSQV, they were not considered positive because the titers obtained were below the entry dilution (1:80) in both PRNT_90_ and PRNT_50_

^b^Considered monotypic for ILHV because the titer was higher than the entry dilution (1:80) in the PRNT_50_

No ZIKV, DENV-1, DENV-3, and DENV-4-specific neutralizing antibodies could be confirmed by PRNT_90_. One black howler monkey serum previously IFA-reactive for all tested flavivirus showed monotypic PRNT_90_ endpoint titers for WNV (16.7%; 95% CI, 0.4–64.1), while another serum previously reactive in the IFA at the dilution 1:100 for ZIKV showed monotypic PRNT_90_ endpoint titers for ILHV (16.7%; 95% CI, 0.4–64.1), whereas one black-handed spider monkey serum that had previously been IFA-reactive for all tested flaviviruses presented monotypic endpoint titers for DENV-2 (4.2%; 95% CI, 0.1–21.1) (Table 2). The difference in the results of these two tests (IFA and PRNT) is likely due to the lower IFA specificity due to flavivirus antibody cross-reactivity [24]. The absence of ZIKV-specific antibodies is in agreement with previous studies from Brazil showing the absence of ZIKV in NHP in areas with reported high ZIKV dispersion in the human population [21, 25]. However, recent reports of potential African lineage ZIKV in Brazilian NHP may suggest that continuous surveillance for ZIKV in NHP is required [26]. The presence of DENV-specific antibodies in New World NHP has been reported in different countries, such as Brazil, Argentina, and Puerto Rico, without evidence of sylvatic transmission cycles [27]. The low DENV seroprevalence and low endpoint titer of 38.2 (Table 2) was compatible with sporadic infection of a captive NHP in a highly endemic area [27]. The presence of neutralizing antibodies against WNV and ILHV in this study should be interpreted cautiously because of cross-reactivity against potentially co-circulating flaviviruses from the same serogroups, such as Saint Louis encephalitis virus (SLEV) and Rocio virus (ROCV) (Fig. 1A), which cannot be excluded even when using highly specific PRNT_90_.

Our serological data may support the occurrence of YFV in NHP in Mexico. On the one hand, we cannot exclude that antigenically related flaviviruses could also have elicited cross-reactive antibodies. This hypothesis is compatible with the detection of a monotypic neutralizing antibody response against Wesselsbron virus in a single Brazilian cow during a sero-epidemiological investigation of several thousand sera from livestock and pets [28]. On the other hand, no flavivirus belonging to the yellow fever serogroup beyond YFV (e.g., Banzi, Bouboui, Jugra, Potiskum, Saboya, Sepik and Wesselsbron viruses) has ever been reported in the Americas, the aforementioned viruses are all endemic to Africa and Asia [28–31]. Additionally, the three YFV-seropositive individuals presented neutralizing antibodies titers either monotypic or higher than fourfold against the other flavivirus serocomplexes, which is deemed highly robust and decisive for flavivirus serological diagnosis [32]. Moreover, the seropositive animals lived in captivity for years within a zoo in Tabasco City within a colony established 30 years ago that was founded by animals seized in the same area. Therefore, despite the fact that the distribution of black-handed spider monkey extends from southeastern Mexico to Panama in which yellow fever may occur, this colony has not been subject to introduction of animals from other countries potentially infected with yellow fever prior to arrival in Mexico. This is relevant because, if confirmed, the presence of YFV would complete the ecological determinants necessary for establishing a sylvatic cycle of yellow fever in southeastern Mexico, i.e., the presence of susceptible wild reservoirs and the presence of invertebrate vectors.

The apparent absence of yellow fever northwards of Panama is not entirely understood. In contrast to other Latin American countries, such as Brazil, in which yellow fever outbreaks often occur, wild NHP populations in Mexico are geographically limited and in small numbers. That is probably one of the reasons because, at least so far, no recent human cases have been reported in Mexico. If YFV was indeed present in Mexico, it would be possible that at any time, YFV may emerge in humans or NHPs.

Our study is chiefly limited by the small number of NHP that were available for testing and the absence of testing for all potentially co-circulating flaviviruses. However, the latter is most critical for WNV, ILHV and BSQV (Fig. 3A) due to likely SLEV co-circulation in Mexico [33] and potential circulation of other flaviviruses reported in Central and South America [32, 34, 35]. In contrast, we covered all circulating serocomplexes and high neutralizing antibody titers and the absence of co-circulating viruses of the same serogroup support robustness of the data for YFV.

## Conclusions

Our findings warrant monitoring of potential YFV infections in free-living and captive NHP as well as mosquito surveillance in Mexico and neighboring countries. Additional confirmation, including direct viral detection, is needed to provide definite assessments of the potential occurrence of YFV in areas north of Panama considered free of yellow fever.

## Data Availability

Materials and data supporting our findings and conclusions are included in the article. The raw data will be made available by the authors, upon request.

## Abbreviations

DENV: Dengue virus
YFV: Yellow fever virus
BSQV: Bussuquara virus
ILHV: Ilhéus virus
SLEV: Saint Louis encephalitis virus
ROCV: Rocio virus
ZIKV: Zika virus
WNV: West Nile virus
NHP: Non-human primates
IFA: Immunofluorescence assay
PRNT: Plaque reduction neutralization test
PAHO: Pan American Health Organization (PAHO)
DMEM: Dulbecco's Modified Eagle Medium
RT-PCR: Reverse transcription polymerase chain reaction
RNA: Ribonucleic acid
